# Overexpression of cyclin‐dependent kinase 1 in esophageal squamous cell carcinoma and its clinical significance

**DOI:** 10.1002/2211-5463.13306

**Published:** 2021-10-19

**Authors:** Han‐Jie Zhang, Gang Chen, Shang‐Wei Chen, Zong‐Wang Fu, Hua‐Fu Zhou, Zhen‐Bo Feng, Jun‐Xian Mo, Chang‐Bo Li, Jun Liu

**Affiliations:** ^1^ Department of Pathology The First Affiliated Hospital of Guangxi Medical University Nanning China; ^2^ Department of Cardio‐Thoracic Surgery The First Affiliated Hospital of Guangxi Medical University Nanning China; ^3^ Department of Cardio‐Thoracic Surgery The Seventh Affiliated Hospital of Guangxi Medical University Wuzhou China; ^4^ Wuzhou Gongren Hospital Wuzhou China

**Keywords:** cyclin dependent kinase 1, esophageal squamous cell carcinoma, immunohistochemistry, RNA‐seq

## Abstract

Cyclin‐dependent kinase 1 (CDK1) plays a significant role in certain malignancies. However, it remains unclear whether CDK1 plays a role in esophageal squamous cell carcinoma (ESCC). The aim of this study was to analyze the expression and clinical value of CDK1 in ESCC. CDK1 protein in 151 ESCC tissues and 138 normal esophageal tissues was detected by immunohistochemistry. RNA‐seq of eight pairs of ESCC and adjacent esophageal specimens was performed to evaluate the levels of *CDK1* mRNA. Microarray and external RNA‐seq data from 664 cases of ESCC and 1733 cases of control tissues were used to verify the difference in CDK1 expression between the two groups. A comprehensive analysis of all data was performed to evaluate the difference in CDK1 between ESCC tissues and control tissues. Further, functional enrichment analyses were performed based on differentially expressed genes (DEGs) of ESCC and co‐expressed genes (CEGs) of CDK1. In addition, a lncRNA‐miRNA‐*CDK1* network was constructed. The expression of CDK1 protein was obviously increased in ESCC tissues (3.540 ± 2.923 vs. 1.040 ± 1.632, *P* < 0.001). RNA‐seq indicated that the mRNA level of CDK1 was also highly expressed in ESCC tissues (5.261 ± 0.703 vs. 2.229 ± 1.161, *P* < 0.0001). Comprehensive analysis revealed consistent up‐regulation of CDK1 (SMD = 1.41; 95% CI 1.00–1.83). Further, functional enrichment analyses revealed that the functions of these genes were mainly concentrated in the cell cycle. A triple regulatory network of PVT1‐hsa‐miR‐145‐5p/hsa‐miR‐30c‐5p‐*CDK1* was constructed using *in silico* analysis. In summary, overexpression of CDK1 is closely related to ESCC tumorigenesis.

## Graphical abstract

Cyclin‐dependent kinase 1 (CDK1) plays a significant role in certain malignancies. The main objectives of this study are to comprehensively evaluate the expression pattern of CDK1 in ESCC and to explore the pivotal regulatory role of CDK1 in ESCC by DEGs, CEGs, lncRNAs, and miRNAs.

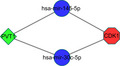

**Abbreviations**
CDKs: cyclin‐dependent kinases
CDK1: cyclin‐dependent kinase 1
CEGs: co‐expressed genes
DEGs: differentially expressed genes
ESCC: esophageal squamous cell carcinoma

Esophageal cancer is one of the most common malignant tumors in the world and is the seventh highest cause of cancer‐related death [1]. There are approximately 19,260 new cases and 15,530 deaths each year [1]. Esophageal squamous cell carcinoma (ESCC) is the main subtype of esophageal cancer, accounting for 90% of all esophageal cancers [2]. Thus far, the main treatment method for ESCC is a combined treatment of radiotherapy, chemotherapy, and surgery [3]. The emergence of drug resistance in molecular targeted therapy leads to poor clinical efficacy in patients with ESCC [4, 5, 6, 7]. According to statistics, from 2010 to 2016, the 5‐year survival rate remains poor (20%) [1], and the mortality rate has remained high [8, 9, 10]. There are still no targeted drugs approved for ESCC, which is still an ongoing research area. Therefore, it is necessary to find a more effective molecular target to obtain a better likelihood of survival.

The family of cyclin‐dependent kinases (CDKs) plays an important role in the major events that drive the cell cycle in eukaryotic cells [11]. The tumor‐related cell cycle is usually regulated by the activity of CDKs [12]. Therefore, it is necessary to understand the principles of the cell cycle for cancer treatment. Cyclin‐dependent kinase 1 (CDK1) is a key gene of mitosis and plays a key role in cell cycle control [13]. According to a report, the up‐regulation of CDK1 can promote the growth and the proliferation of melanoma tumor cells. They found in *in vivo* experiments that the tumor volume increased significantly in overexpressed wild‐type CDK1 cells compared with vector control or kinase‐dead variant CDK1 cells [14]. Moreover, studies have shown that the overexpression of CDK1 in colorectal cancer [15, 16], and the authors found that the decreased proliferation of colorectal cancer cells after NFE2L3 knockout may be due to the decreased CDK1 activity [15].

In terms of the expression patterns of CDK1 in esophageal cancer, the importance of CDK1 has been demonstrated in some studies. For example, a research team previously confirmed the overexpression of CDK1 in 59 cases of esophageal cancer using real‐time polymerase chain reaction and Western blot [17]. They found that in normal esophageal mucosa, CDK1 was located in the basal layer of the squamous epithelium [17]. Unfortunately, the team only focused on esophageal adenocarcinoma and did not mention the results of ESCC. Meanwhile, Bao‐Ai Han *et al*. (2020) [18] reported that CDK1 is overexpressed in ESCC tissues according to Human Protein Atlas (HPA) and The Cancer Genome Atlas (TCGA). The disadvantage is that they only obtained the mRNA support of *CDK1* from the TCGA database and did not conduct experiments to verify the protein level and mRNA expression level of *CDK1*. Further, the correlation between CDK1 and ESCC has also been verified. Shen‐bo Fu *et al*. (2018) [19] reported that the sinomenine hydrochloride can enhance the radiosensitivity of ESCC cells, which might be related to the down‐regulation of CDK1. Unfortunately, they did not report the expression of *CDK1* mRNA. Hence, our study integrates a variety of data—including immunohistochemistry data, in house RNA‐seq data, microarray, and external RNA‐seq data—to comprehensively analyze the expression significance of CDK1 in ESCC.

According to the data from TCGA database, the low expression of *CDK1* was associated with a worse relapse‐free survival rate in ESCC patients [20]. In addition, Mingyao Li *et al*. (2020) analyzed the prognostic value of *CDK1* mRNA expression in lung cancer patients using KM plotter. However, the result of Mingyao Li *et al*. (2020) [21] is inconsistent with that of Dong *et al*. (2018) [20]. Lung cancer patients with high expression of *CDK1* have worse overall survival [21]. Therefore, it is necessary to use a large amount of data to verify the expression and clinical value of CDK1 in ESCC from various aspects and explore its regulatory role in ESCC from different perspectives.

Further, gene expression is regulated by non‐coding RNA, particularly long noncoding RNA (lncRNA) and microRNA (miRNA). The competitive endogenous RNA (ceRNA) hypothesis suggests that the regulation between non‐coding RNAs and mRNAs is achieved by competitive binding to shared miRNAs. In recent years, an increasing number of studies have revealed that the lncRNA‐miRNA‐mRNA network plays a key role in the progression and metastasis of various cancers, such as pancreatic cancer [22], liver cancer [23], and osteosarcoma [24]. One study has revealed that the MMP9/ITGB1‐miR‐29b‐3p‐HCP5 is related to the prognosis of pancreatic cancer [25]. However, the mechanism of the lncRNA‐miRNA‐mRNA network, which regulates the expression of *CDK1* in ESCC, remains unclear. Therefore, it is necessary to study the regulatory relationship between *CDK1* and its upstream lncRNAs and miRNAs in order to identify the mechanism of CDK1 in ESCC.

The main objectives of this study are to comprehensively evaluate the expression pattern of CDK1 in ESCC and to explore the pivotal regulatory role of CDK1 in ESCC by differentially expressed genes (DEGs), co‐expressed genes (CEGs), lncRNAs, and miRNAs. First, we evaluated the clinical significance of CDK1 in ESCC from protein (immunohistochemistry) and mRNA levels (in‐house RNA‐seq and external RNA‐seq data and microarray data). In addition, all data were integrated for a comprehensive analysis to evaluate the clinical value of CDK1 expression. Second, the regulatory role of CDK1 in the cell cycle of ESCC was analyzed by the DEGs and CEGs of CDK1. By predicting the miRNAs and lncRNAs upstream of *CDK1*, we constructed a ceRNA network to demonstrate the regulatory relationship between *CDK1* and lncRNAs and miRNAs upstream, which further demonstrated the regulatory role of CDK in ESCC.

## Results

### Process of this paper

The process flowchart of this paper is illustrated in Fig. S1.

### Overexpression of CDK1 protein in ESCC

**Fig. 1:**
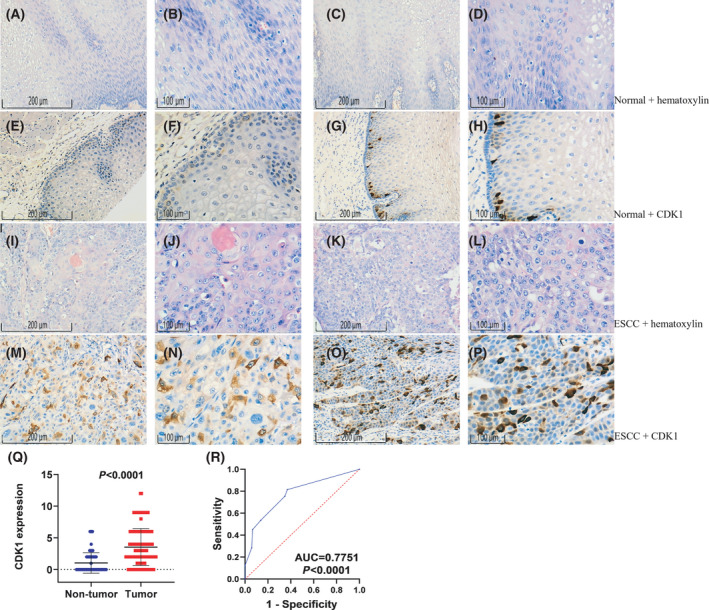
Expression of CDK1 protein in ESCC and normal tissues in tissue microarray: hematoxylin staining in normal tissues (A–D; 100x, 200x); CDK1 protein expression in normal tissues (E–H; 100x, 200x); hematoxylin staining in ESCC tissues (I–L; 100x, 200x); CDK1 protein expression in ESCC tissues (M–P; 100x, 200x); Scatter plot and ROC curve (Q, R). Note: The statistical comparison method was Student's *t* test. The measuring scale bars = Ratio of distance on the graph to actual distance. In other words, the distance of 100 μm in the figure is actually 1 μm, and the distance of 200 μm is actually 2 μm.

According to the immunohistochemical results of 289 samples, compared with normal esophageal tissues, the total score of CDK1 staining intensity and cell percentage in ESCC tissues was obviously higher (3.540 ± 2.923 vs. 1.040 ± 1.632, *P* < 0.001; Fig. 1Q, Table S1). The immunohistochemical staining of CDK1 was mainly in the nucleus and cytoplasm (Fig. 1A–P). The area under curve (AUC) was 0.775 (95% CI 0.7451–0.8685, *P* < 0.0001) and the sensitivity and specificity were 0.815 and 0.630, respectively (Fig. 1R). In addition, the correlation between the expression of CDK1 and different gender, age, TNM staging, and lymph node metastasis failed to find significant differences (Table S1).

### In‐house RNA‐seq level

**Fig. 2:**
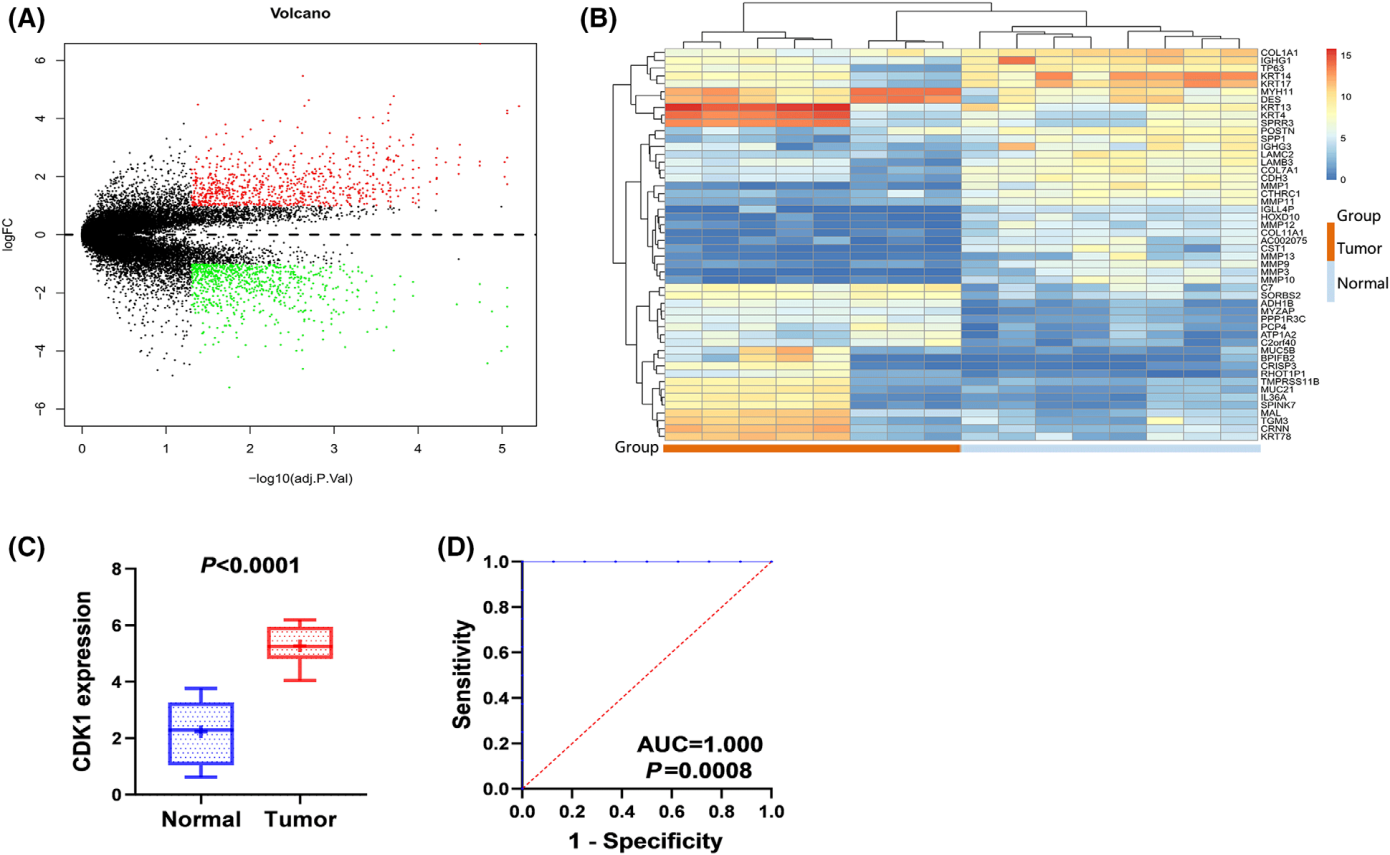
Over‐expression of the *CDK1* mRNA level in ESCC tissues based on in‐house RNA‐seq data. (A) DEGs volcano map; (B) DEGs heat map; (C) Boxplot of in‐house RNA‐seq calculated by Student's *t* test; (D) In house RNA‐seq ROC curve.

To verify the overexpression of *CDK1* in ESCC tissues, eight pairs of ESCC samples and control samples were used for RNA‐seq data of *CDK1*. The result indicated that there was obvious difference in mRNA expression of *CDK1* between cancer tissues and normal esophageal tissues (Fig. 2A–D), and the expression of *CDK1* was obviously increased in ESCC tissues (5.261 ± 0.703 vs. 2.229 ± 1.161, *P* < 0.0001; Fig. 2C). These results further confirmed the overexpression of *CDK1* in ESCC tissues. In addition, the ROC analysis revealed that *CDK1* can clearly distinguish ESCC tissues from normal esophageal tissues (AUC ˜ 1, *P* = 0.0008; Fig. 2D).

### mRNA expression level of external RNA‐seq data and microarray data

**Fig. 3:**
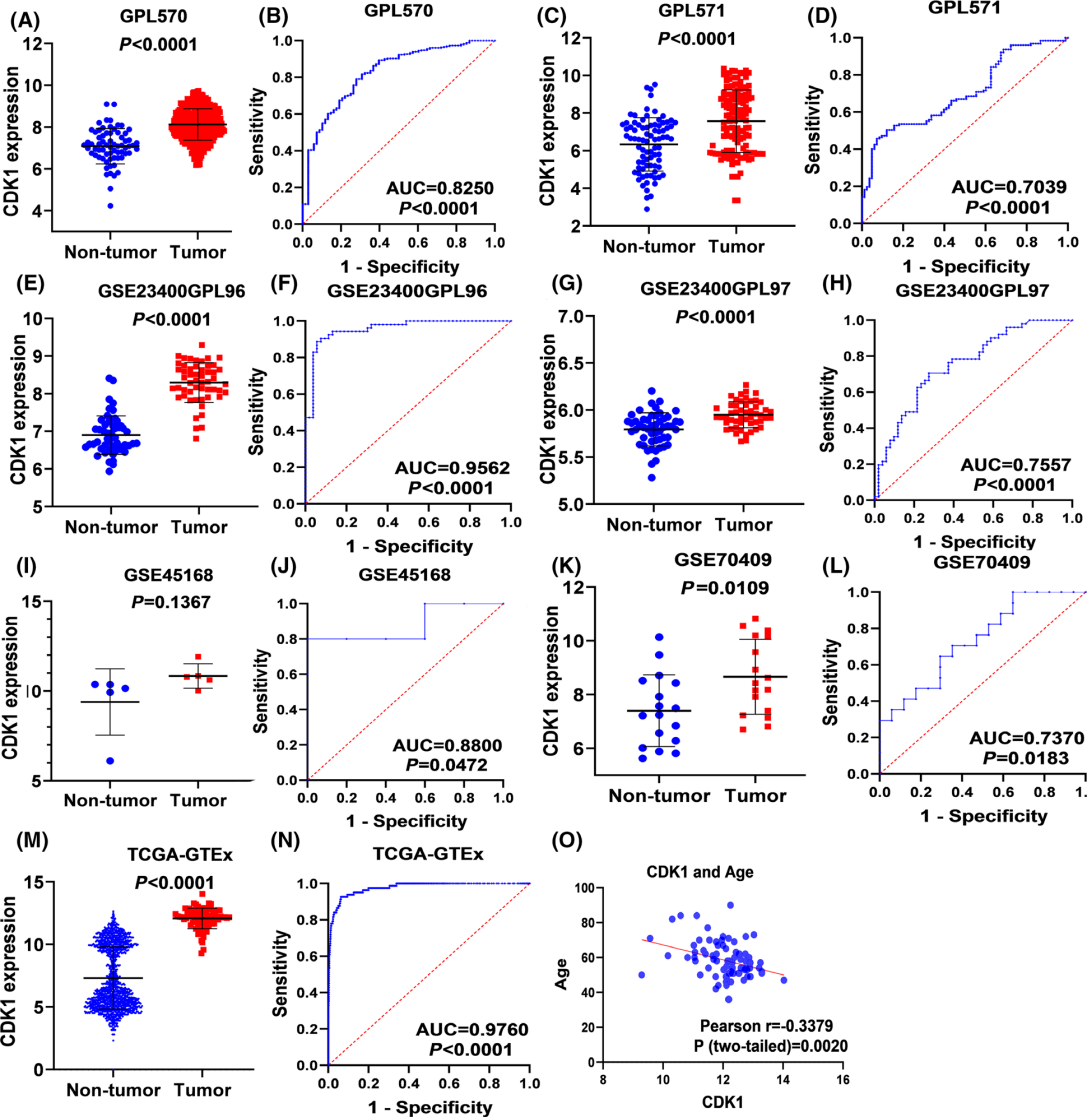
Over‐expression of *CDK1* in ESCC in microarray and external RNA‐seq data. GPL570 (A, E); GPL 571 (B, F); GSE23400GPL96 (C, G); GSE23400GPL97 (D, H); GSE45168 (I, K); GSE70409 (J, L); TCGA‐GTEx (M, N); Pearson correlation analysis of CDK1 expression and age (O). Note: The statistical comparison method was Student's *t* test.

After eliminating the batch effect and integrating the data from the same platform, seven expression data sets (including GPL570, GPL571, GSE23400, GSE32424, GSE45168, GSE70409, and TCGA‐GTEx) of the ESCC and normal group were collected. The basic information of microarray data was presented in Table 1. By comparison, we found that there was an obvious difference in the expression level of *CDK1* between two groups. According to the scatter plot (Fig. 3A–O), except for GSE45168, the mRNA value of *CDK1* in the ESCC group was obviously higher than that in the control group. As is evident from the ROC curve (Fig. 3), these data sets all indicated that *CDK1* expression has a strong ability to identify ESCC and normal tissues. In addition, we also estimated the correlation between *CDK1* expression and clinicopathological parameters of ESCC (Table S2). The results indicated that the mRNA level of *CDK1* in patients with ESCC in different age groups was obviously different (*P* = 0.001). Pearson’s correlation analysis indicated that age was negatively correlated with the expression level of *CDK1*, thereby indicating that the younger the patients, the higher the expression level of *CDK1* (*R* = −0.338, *P* < 0.05). However, the expression of *CDK1* was not significantly correlated with gender (*P* = 0.275), T phase (*P* = 0.545), N phase (*P* = 0.423), M stage (*P* = 0.714), and drinking (*P* = 0.812).

**Table 1:** Basic information of microarray data.

Author	Year	Country	Data source	Platform	Sample type	RNA type
Wen J *et al*.	2014	China	GSE45670	Affymetrix GPL570	Tissue	mRNA/lncRNA
Erkizan HV *et al*.	2017	USA	GSE77861	Affymetrix GPL570	Tissue	mRNA/lncRNA
Wang Q *et al*.	2013	Germany	GSE26886	Affymetrix GPL570	Tissue	mRNA/lncRNA
Tanaka Y *et al*.	2017	Japan	GSE69925	Affymetrix GPL570	Tissue	mRNA/lncRNA
Ming XY *et al*.	2017	China	GSE100942	Affymetrix GPL570	Tissue	mRNA/lncRNA
Chen K *et al*.	2011	China	GSE33810	Affymetrix GPL570	Tissue	mRNA/lncRNA
Lee JJ *et al*.	2009	USA	GSE17351	Affymetrix GPL570	Tissue	mRNA/lncRNA
Hu N *et al*.	2011	USA	GSE20347	Affymetrix GPL571	Tissue	mRNA/lncRNA
Hu N *et al*.	2015	USA	GSE38129	Affymetrix GPL571	Tissue	mRNA/lncRNA
Yan W *et al*.	2011	USA	GSE29001	Affymetrix GPL571	Tissue	mRNA/lncRNA
Yan W *et al*.	2011	USA	GSE33426	Affymetrix GPL571	Tissue	mRNA/lncRNA
Su H *et al*.	2010	USA	GSE23400	Affymetrix GPL96/GPL97	Tissue	mRNA/lncRNA
Tong M *et al*.	2012	USA	GSE32424	Illumina GPL10999	Tissue	mRNA/lncRNA
Zhu L *et al*.	2013	China	GSE45168	Agilent GPL13497	Tissue	mRNA/lncRNA
Chen YK *et al*.	2015	China	GSE70409	Phalanx GPL13287	Tissue	mRNA/lncRNA
Cao W *et al*.	2013	Canada	GSE45350	Agilent GPL13607	Tissue	mRNA/lncRNA
Chen Z *et al*.	2014	China	GSE43732	Agilent GPL16543	Tissue	miRNA
Wen J *et al*.	2018	China	GSE114110	Agilent GPL24967	Tissue	miRNA
Shi R *et al*.	2015	China	GSE59973	Agilent GPL16770	Tissue	miRNA
Guo Y *et al*.	2012	China	GSE6188	Tsinghua University GPL4508	Tissue	miRNA
Jang HJ *et al*.	2017	USA	GSE55856	Affymetrix GPL14613	Tissue	miRNA
Lin C *et al*.	2017	China	GSE97049	Affymetrix GPL21572	Tissue	miRNA
Liao J *et al*.	2015	China	GSE71043	Agilent GPL18402	Blood	miRNA
Zheng D *et al*.	2019	China	GSE112840	Agilent GPL23365	Serum	miRNA

### Comprehensive analysis results of all data sets

**Fig. 4:**
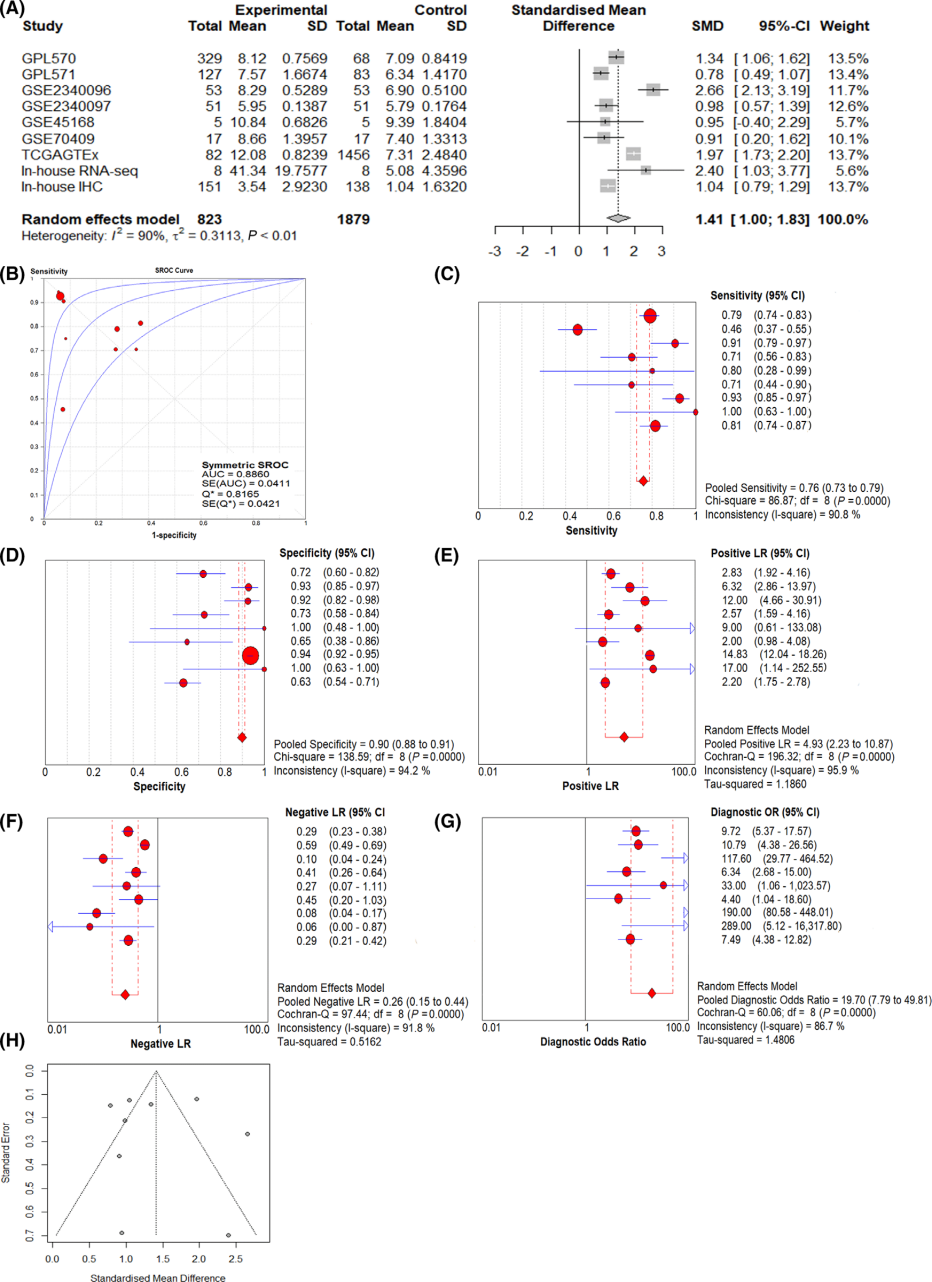
Comprehensive analysis of over‐expression of *CDK1* in ESCC tissues based on all data. (A) SMD forest map; (B) SROC curve; (C) Sensitivity; (D) Specificity; (E) Positive likelihood ratio; (F) Negative likelihood ratio; (G) Diagnostic odds ratio; (H) Funnel plot. Note: The red dots represent the effect quantity of a single study, the diamonds represent the combined effect quantity, and the arrows represent that the confidence interval of the effect quantity is beyond the set range.

To improve the reliability of the results of this research, we integrated all the data for a comprehensive analysis (including in‐house IHC, in‐house RNA‐seq, and microarray and external RNA‐seq data). The forest map revealed that CDK1 expression was significantly up‐regulated in ESCC (SMD = 1.41; 95% CI 1.00–1.83; Fig. 4A), the AUC was 0.886 (Fig. 4B), and the sensitivity and specificity of combination were 0.760 (95% CI: 0.73–0.79) and 0.900 (95% CI: 0.88–0.91), respectively (Fig. 4C,D). The positive likelihood ratio was 4.930 (95% CI = 2.23‐10.87; *I* ^2^ = 95.9% Fig. 4E), and the negative likelihood ratio was 0.260 (95% CI = 0.15‐0.44; I^2^ = 91.8% Fig. 4F). The diagnostic OR was 19.700 (95% CI = 7.79–49.8; I^2^ = 86.7% Fig 4G). The funnel plot (Fig. 4H) indicates no published bias in these data sets (For Begg’s test *P* = 0.754, and that of Egger’s test was 0.810).

### The expression of *CDK1* was verified by cells data

**Fig. 5:**
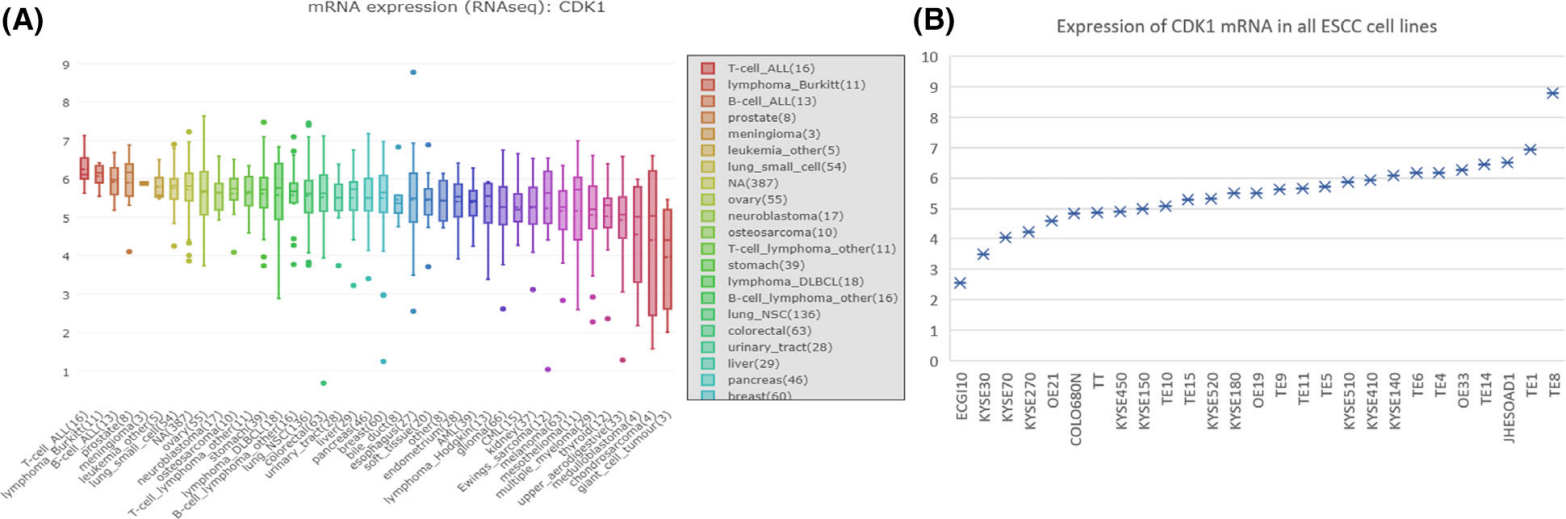
Over‐expression of *CDK1* in the Cancer Cell Line Encyclopedia (CCLE) cell lines. (A) The expression level of *CDK1* in all cell lines; (B) CDK1 in all ESCC cell lines.

The mRNA expression profiles from the CCLE database indicated that the mRNA level of *CDK1* in ESCC cell lines was higher than that in most other types of tumor cells, including prostate tumor, lung small‐cell cancer, stomach tumor, colorectal tumor, etc. (Fig. 5A,B). The scatter plot of *CDK1* expression in multifarious ESCC cell lines (KYSE450, TE10, TE6, TE4, ECGI10, TE14, TE8, OE33, KYSE410, KYSE140, KYSE180, TE9, KYSE520, KYSE270, TT, KYSE70, TE11, TE1, TE5, TE15, OE19, KYSE510, KYSE30, KYSE150, JHESOAD1, COLO680N, OE21) revealed that the expression of *CDK1* was at a high level.

### Molecular mechanism of CDK1 and its CEGs in ESCC

**Fig. 6:**
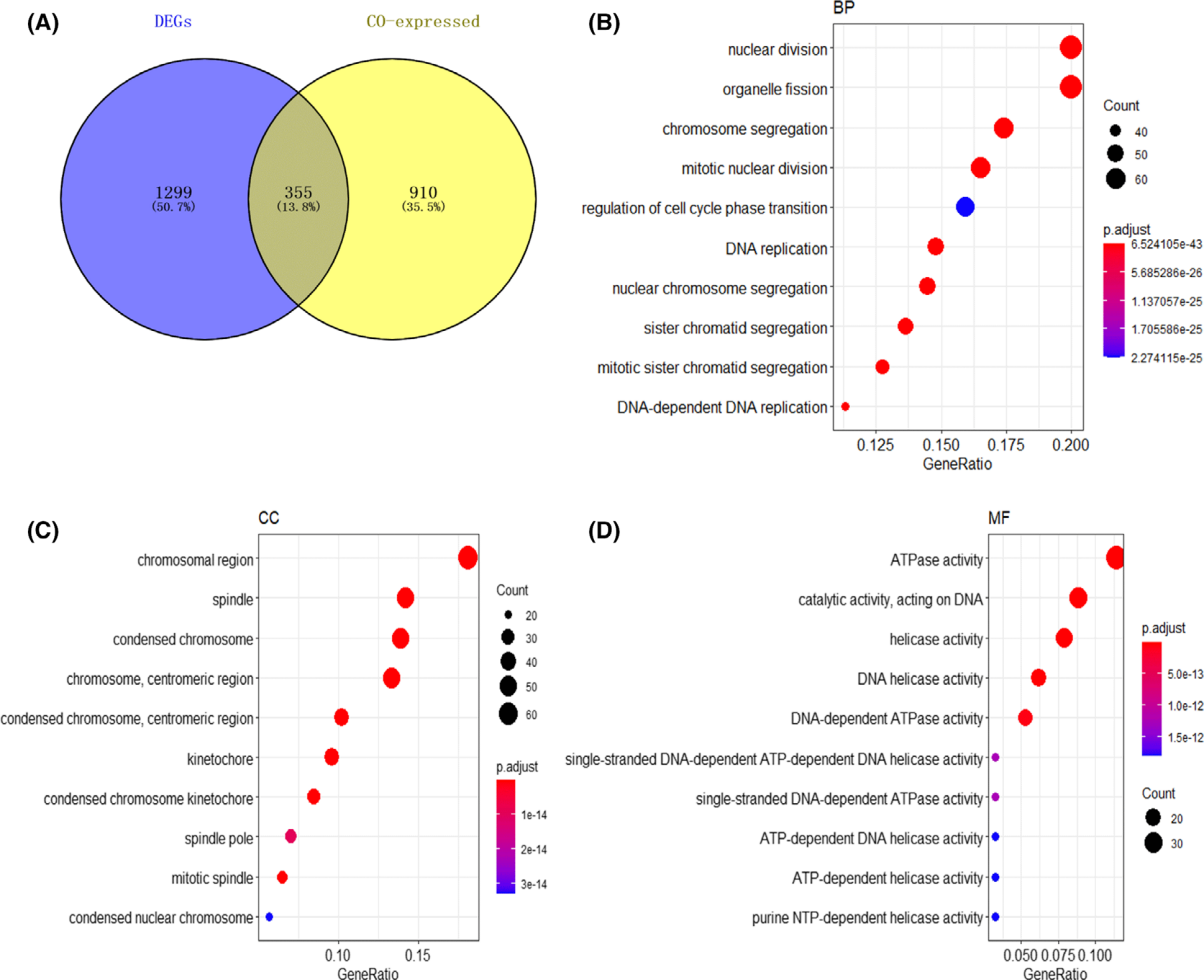
GO functional annotation on the possible molecular mechanisms of CDK1 in ESCC based on 355 overlapping genes of DEGs and CEGs. (A) Venn diagram; (B) Biological process; (C) Cell composition; (D) Molecular function. Note: GO, gene ontology; DEGs, differentially expressed genes; CEGs, co‐expressed genes.

To study the molecular mechanism of CDK1 in ESCC, 23,872 DEGs (including 14,594 up‐regulated genes and 9278 down‐regulated genes) and 11,450 CEGs associated with CDK1 were screened by R3.6.3. The DEGs of the in‐house cohort was shown in Table S3. In order to screen more reliable genes, 355 co‐expressed DEGs were obtained by overlapping DEGs with CEGs in three or more data sets (Fig. 6A). GO functional enrichment and KEGG pathway analysis were performed on these overlapping CEGs. As expected, in the biological process (BP), it is mainly enriched in nuclear division, organelle fission, and chromosome segregation (Fig. 6B). In the cellular component (CC), it is mainly concentrated in the chromosome region, spindle, and condensed chromosome (Fig. 6C). In terms of molecular function (MF), it is mainly enriched in ATPase activity, catalytic activity, acting on DNA, and helicase activity (Fig. 6D).

**Fig. 7:**
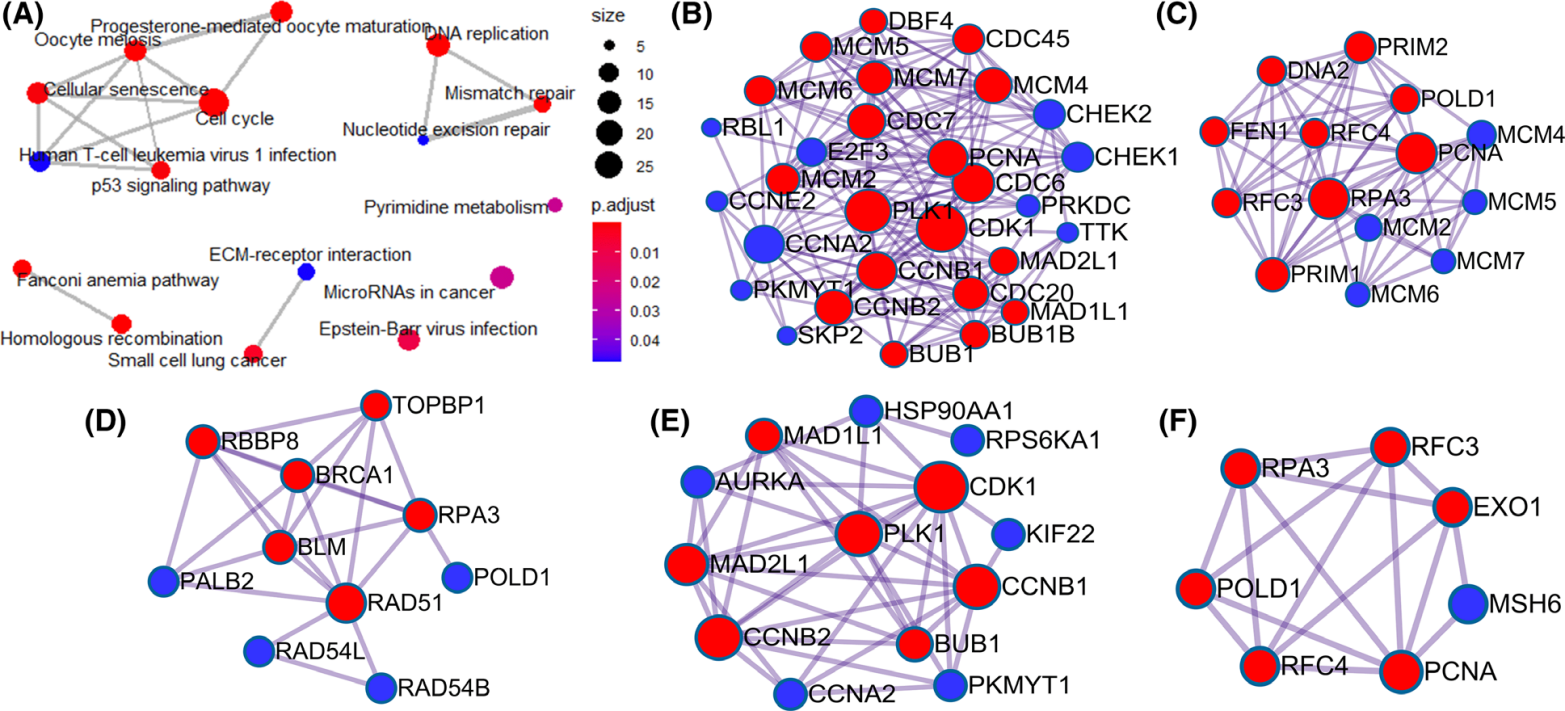
KEGG pathway on the possible molecular mechanisms of CDK1 in ESCC based on 355 overlapping genes of DEGs and CEGs. (A) KEGG pathway analysis; PPI network of genes enriched in the first five KEGG pathways: (B) Cell cycle; (C) DNA replication; (D) Homologous recombination; (E) Progesterone‐mediated oocyte maturation; (F) Mismatch repair. Note: KEGG, Kyoto Encyclopedia of Genes and Genomes; PPI, protein interaction network; DEGs, differentially expressed genes; CEGs, co‐expressed genes.

Interestingly, in KEGG pathway analysis, 355 co‐expressed DEGs are mainly concentrated in the cell cycle, in addition to DNA replication, homologous recombination, progesterone‐mediated oocyte maturation, mismatch repair, etc. (Fig. 7A). These results suggested that CDK1 may regulate numerous biological functions in the pathogenesis of ESCC by affecting cell cycle and cell division. Considering that proteins need to interact with each other to play biological roles, we selected genes enriched in the first five pathways of KEGG to construct the PPI network (Fig. 7B–F). The results revealed that there were 19 hub genes interacting closely in the cell cycle pathway—CCNB2, CCNB1, CDC7, DBF4, MCM6, BUB1, PCNA, CDC20, CDC45, MCM2, MCM4, BUB1B, MAD1L1, PLK1, MCM7, MAD2L1, CDK1, CDC6, MCM5. Further, there were nine hub genes in DNA replication pathways (FEN1, PCNA, PRIM1, RFC3, RPA3, PRIM2, POLD1, RFC4, and DNA2), seven hub genes in homologous recombination pathways (PALB2, RBBP8, RPA3, BRCA1, BLM, RAD51, and TOPBP1), seven hub genes in progesterone‐mediated oocyte maturation pathways (CDK1, BUB1, MAD1L1, PLK1, CCNB2, CCNB1, and MAD2L1), and six hub genes in mismatch repair pathways (RFC3, PCNA, EXO1, RFC4, POLD1, and RPA3). These results suggested that CDK1 may interact with these proteins to influence cell cycle and DNA replication and promote the occurrence and development of ESCC.

### Construction of the ceRNA network

**Fig. 8:**
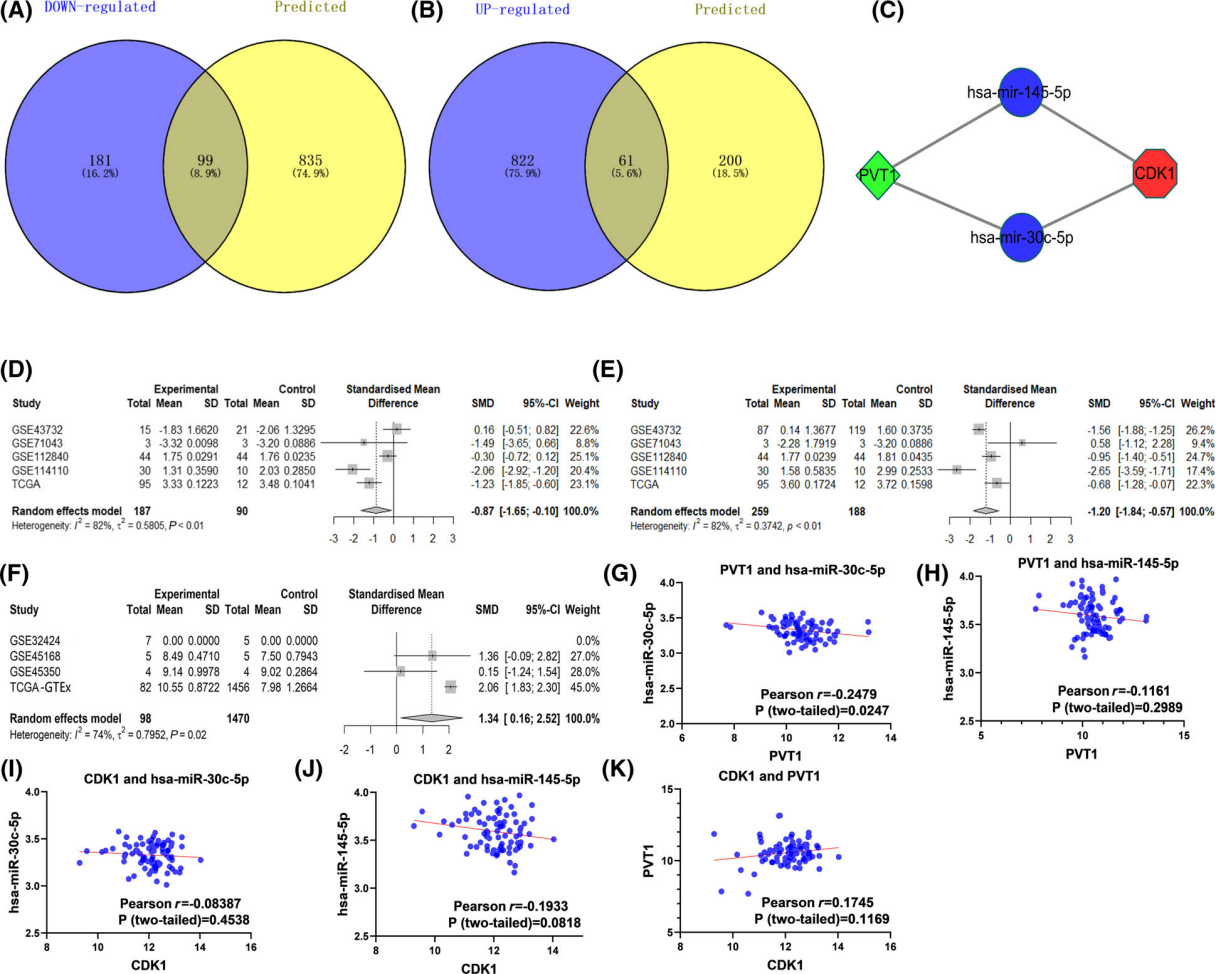
Constructed ceRNA network. (A) Venn diagram of down‐regulated miRNA and predicted miRNA; (B) Venn diagram of up‐regulated lncRNA and predicted lncRNA. (C) The lncRNA‐miRNA‐CDK1 ceRNA network. (D) Hsa‐miR‐30c‐5p SMD forest map; (E) hsa‐miR‐145‐5p SMD forest map; (F) PVT1 SMD forest map; (G) PVT1 and hsa‐miR‐30c‐5p Pearson correlation analysis; (H) PVT1 and hsa‐miR‐145‐5p Pearson correlation analysis; (I) CDK1 and hsa‐miR‐30c‐5p Pearson correlation analysis; (J) CDK1 and hsa‐miR‐145‐5p Pearson correlation analysis; (K) CDK1 and PVT1 Pearson correlation analysis. Note: ceRNA, endogenous competitive RNA network.

According to the ceRNA theory, lncRNA functions as a miRNA sponge to inhibit the function of miRNA, and the expression level of miRNA is negatively correlated with mRNA and lncRNA. First, 934 miRNAs upstream of *CDK1* were obtained from the miRwalk website and overlapped with 280 down‐regulated miRNAs (Fig. 8A); in addition, the SMD and AUC of 99 overlapping miRNAs was calculated. Finally, 22 miRNAs with SMD < 0 and *P* < 0.05 were obtained (Table 1). These 22 miRNAs were input into miRNet database to predict 261 upstream lncRNAs. Similarly, 261 lncRNAs were crossed with 1943 up‐regulated lncRNAs and, finally, we obtained 61 overlapping lncRNAs (Fig. 8B). The SMD and AUC of these 61 overlapping lncRNAs was calculated and we finally obtained PVT1, a most significantly up‐regulated lncRNA (PVT1, SMD = 1.340; 95% CI 0.16–2.52, AUC = 0.915; Table 2). According to the above account, we constructed the lncRNA‐miRNA‐mRNA (PVT1‐hsa‐miR‐145‐5p/hsa‐miR‐30c‐5p‐*CDK1*) network (Fig. 8C). All lncRNAs and miRNAs, miRNAs and *CDK1* were consistent with the principle of ceRNA network hypothesis. According to Pearson’s correlation analysis, the expression of PVT1 and hsa‐miR‐30c‐5p was negatively correlated (*R* = −0.248, *P* = 0.0247). Although the correlation analysis of miRNAs and lncRNAs expression failed to find statistical significance, our comprehensive SMD calculation results revealed that both PVT1 and *CDK1* indicated opposite expression patterns with hsa‐miR‐145‐5p (SMD = −1.200; 95% CI −1.84 to −0.57, AUC = 0.913) and hsa‐miR‐30c‐5p (SMD = 0.870; 95% CI: −1.55 to −0.10, AUC = 0.858; Figs. 8D–K). This sub‐network may also provide new directions for research on the molecular mechanisms of ESCC.

**Table 2:** SMD and AUC of miRNAs and lncRNAs.

ID	Tumor total *N*	Non‐tumor total *N*	SMD	95%Cl	AUC
hsa‐miR‐1	187	301	−1.290	−2.01; −0.56	0.859
hsa‐miR‐10a‐3p	142	59	−0.770	−1.19; −0.35	0.919
hsa‐miR‐1244	155	155	−0.940	−1.17; −0.70	0.825
hsa‐miR‐135a‐5p	108	59	−1.030	−1.46; −0.59	0.927
hsa‐miR‐145‐3p	146	69	−0.840	−1.22; −0.46	0.842
hsa‐miR‐145‐5p	259	188	−1.060	−1.95; −0.16	0.913
hsa‐miR‐155‐3p	103	51	−0.940	−1.37; −0.51	0.783
hsa‐miR‐204‐5p	130	59	−0.740	−1.16; −0.31	0.901
hsa‐miR‐26b‐3p	142	59	−0.680	−1.17; −0.19	0.939
hsa‐miR‐30a‐3p	259	163	−0.830	−1.25; −0.42	0.838
hsa‐miR‐30c‐5p	187	90	−0.870	−1.65; −0.10	0.858
hsa‐miR‐30d‐3p	142	59	−0.740	−1.08; −0.39	0.937
hsa‐miR‐30e‐3p	315	173	−0.550	−0.96; −0.13	0.684
hsa‐miR‐374b‐3p	138	58	−1.100	−1.53; −0.66	0.926
hsa‐miR‐499a‐5p	88	54	−0.990	−0.42; −0.56	0.787
hsa‐miR‐526b‐5p	199	50	−0.560	−0.98; −0.14	0.944
hsa‐miR‐548a‐3p	155	155	−1.620	−2.68; −0.56	0.897
hsa‐miR‐548c‐3p	54	54	−1.080	−1.49; −0.67	0.845
hsa‐miR‐548d‐5p	162	162	−0.610	−1.14; −0.08	0.731
hsa‐miR‐624‐5p	140	58	−0.550	−0.97; −0.13	0.851
hsa‐miR‐628‐5p	142	59	−0.920	−1.28; −0.57	0.845
hsa‐miR‐873‐5p	94	55	−0.590	−1.01; −0.17	0.833
PVT1	159	1793	1.340	0.16; 2.52	0.915

## Discussion

Our study is the first multicenter study to investigate and verify the expression pattern of CDK1 in ESCC. First, we found that the CDK1 protein level was increased in ESCC tissue and then the overexpression of *CDK1* mRNA was confirmed by in‐house RNA‐seq, microarray and external RNA‐seq data. The key genes of CDK1 in ESCC with regard to the cell cycle were obtained by GO and KEGG pathway enrichment and PPI network of co‐expressed DEGs associated with CDK1. In addition, according to the principle of the ceRNA network, we explored lncRNA and miRNA involved in *CDK1* regulation and successfully constructed lncRNA‐miRNA‐mRNA (PVT1‐hsa‐miR‐145‐5p/hsa‐miR‐30c‐5p‐*CDK1*) network.

In this study, we estimated the expression level of CDK1 in terms of various aspects (including immunohistochemistry experiment, in‐house RNA‐seq, external RNA‐seq and microarray data) in a total of 2702 cases, which is unprecedented. In this process, we demonstrated that the expression level of CDK1 was obviously increased in ESCC compared with normal esophageal tissues. In addition, we suggested that CDK1 has relatively high sensitivity and specificity in distinguishing ESCC tissues from normal esophageal tissues. However, in normal esophageal tissues, CDK1 was expressed in basal cells. The changes in expression fully indicated that CDK1 may be involved in the occurrence of ESCC cells. Further, the large number of cases for which we estimated the expression level can improve the reliability of our results. In addition, we also used CCLE esophageal cancer cell lines to confirm the high expression level of *CDK1* mRNA, which indicates that the high expression of *CDK1* is indeed present in esophageal cancer cells rather than in the tumor matrix, but these results must be confirmed by further single‐cell sequencing.

In terms of potential molecular mechanisms, our study provides important clues for the role of CDK1 in ESCC. A previous study has reported that the down‐regulation of CDK1 may be involved in the mechanism of radiosensitivity of ESCC [19]. The researchers found that the radiosensitization effect of sinomenine hydrochloride is related to the down‐regulation of CCNB1 and CDK1 in ESCC cells [19]. Dong *et al*. (2018) [20] revealed that CDK1 may be a regulator of the G2/M pathway of ESCC by functional enrichment analysis and WGCNA. In addition, a few researchers suggested that CDK1 is a key protein in cell cycle regulation and plays a momentous role in cell proliferation [12]. These studies suggested that CDK1 may play a momentous role in the cell cycle during the development of ESCC. However, none of the above studies focused on the CDK1 pathway and its interacting proteins. In this study, by exploring the DEGs and CEGs of CDK1, we found that these genes are mainly concentrated in the cell cycle pathway and the DNA replication pathway. These genes mainly include CCNB2, CCNB1, and CDC7. It is worth noting that the CDK1‐binding compound cyclin B1 (CCNB1) is overexpressed in the tumorigenesis of esophageal cancer [26]. The researchers found that the number of CCNB1 positive cells increased gradually with the degree of esophageal dysplasia from low to high, and the overexpression of CCNB1 in patients with ESCC was also related to the poor prognosis [27]. It has been reported that the down‐regulation of mRNA and protein levels of CCNB1 resulted in G2/M arrest in the cell cycle [28]. Moreover, the active antitumor drug dracorhodin perchlorate (DP) induced the G2/M phase cell cycle arrest of ESCC cells (ECA109, EC9706, and KYSE410) by down‐regulating CCNB1 [29]. The results of the abovementioned two studies are consistent. Moreover, it has been reported that CCNB1 is highly expressed in poorly differentiated ESCC cells and the patients with low CCNB1 expression have better prognosis [30]. In our study, CCNB1 participates in the cell cycle pathway and is one of the hub genes of the PPI network. As mentioned above, CDK1 is a key regulatory gene of the cell cycle. According to a previous study, CDK1 may be a regulator of the G2/M pathway in ESCC cells [20]. The abovementioned studies suggested that the interaction between CDK1 and CCNB1 may have an assignable role in the process of the ESCC cell cycle. For CCNB2, a few researchers have used DEGs to construct a PPI network and found that CCNB2 may participate in the regulation of the tumorigenesis in ESCC [31], which is consistent with the results of our pathway enrichment analysis. With regard to CDC7, it has been reported that CDC7 is obviously up‐regulated in ESCC tissues. The researchers also found that knocking out CDC7 can inhibit proliferation, migration, and invasion of ESCC cells and also induce apoptosis [32]. Our pathway enrichment and PPI network analysis indicate that there is an interaction between CDK1 and CDC7, which indicates that the synergistic effect of CDK1 and CDC7 may play an assignable role in the progress of ESCC. In general, we suppose that CDK1 and these hub genes (CCNB1, CCNB2, and CDC7) are involved in the regulation of tumorigenesis in ESCC. Our study provides information for further study on the pathogenesis of ESCC.

Further, gene expression is regulated by lncRNA and miRNA, which cannot be ignored [33, 34]. Since Salmena *et al*. [35] first proposed the ceRNA hypothesis, an increasing number of researchers are focusing on the role of ceRNA in human cancer and its important role in esophageal cancer has also been reported. For example, a study has indicated that CADM2‐ADAMTS9‐AS2 ceRNA network centered on hsa‐miR‐372, and SERPINE1‐PVT1 ceRNA network centered on hsa‐miR‐145 may be potential carcinogenic mechanisms of esophageal cancer [36]. In addition, it has been reported that the lncRNA‐miRNA‐mRNA ceRNA network, which contains 40 lncRNAs, 28 miRNAs and 233 mRNAs, plays a regulatory role in esophageal cancer [37]. However, the ceRNA regulatory network of *CDK1* in ESCC has not been reported yet. Therefore, we explored the regulatory network of upstream miRNA and lncRNA centered on *CDK1* in ESCC. By further analysis of SMD and Pearson correlation, we successfully constructed two pairs of subnetworks PVT1‐hsa‐miR‐145‐5p / hsa‐miR‐30c‐5p‐*CDK1*. Importantly, the role of these lncRNA and miRNAs in esophageal cancer in this network has been reported in the literature. For example, it has been reported that overexpression of PVT1 is relevant to poor prognosis of esophageal adenocarcinoma [38]. Furthermore, according to the results of miRNA chip and qRT‐PCR analysis, the expression level of miR‐145 decreased in most esophageal cancer samples. They also found that the migration of esophageal cancer cell lines (KYSE150 and KYSE180) transfected with miR‐145 can be inhibited [39]. The expression of miR‐145 was negatively correlated with lymph node metastasis of ESCC [40]. More interestingly, the down‐regulation of PVT1 may inhibit cell migration and invasion by promoting the expression of miR‐145 [41, 42]. These reports further support the reliability of our ceRNA network and indicate that the expression relationship between PVT1 and miR‐145 may play a significant role in the ESCC. Nevertheless, additional experiments are required to confirm these speculations. Although the expression pattern and molecular mechanism of hsa‐miR‐30c‐5p in esophageal cancer has not been reported, its expression pattern in other tumors has been reported. For example, isolinderalactone (a drug) induces apoptosis in breast cancer cells (MDA‐MB‐231) and inhibits the STAT3 signaling pathway by regulating hsa‐miR‐30c‐5p [43]. In other words, these reports support our results to a certain extent.

Based on the reports mentioned above and the results of this study, we hypothesize that the expression of *CDK1* may be regulated by PVT1, hsa‐miR‐145‐5p, and hsa‐miR‐30c‐5p. CDK1 is involved in multifarious biological functions that play important roles in the cell cycle and cell division of ESCC. This *CDK1*‐centered ceRNA network may reveal the potential molecular mechanism of CDK1 regulating the cell cycle of ESCC and provide a new target for the study of ESCC.

However, this study also has a few limitations. In other words, these results require further experimental verification, and further verification of the expression pattern of CDK1 through single‐cell sequencing is necessary.

## Conclusions

To summarize, a comprehensive analysis of the clinical significance of CDK1 expression confirmed that there was an increase in the CDK1 expression in ESCC than that in noncancer cells. In addition, we also constructed a novel lncRNA‐miRNA‐mRNA ceRNA (PVT1‐hsa‐miR‐145‐5p/hsa‐miR‐30c‐5p‐*CDK1*) triple regulatory network. The relationship among these RNAs may provide a few key clues for the molecular mechanism of ESCC in the future.

## Methods

### Tissue microarray (in‐house IHC)

The samples for in‐house IHC were provided by the tissue microarrays (ESC242, ESC1503 and ESC1504) from Pantomics, Inc (Richmond, CA 94806, USA), from January 2015 to December 2018, which included 151 ESCC tissues and 138 normal esophageal tissues, from 156 men and 18 women. All samples were formalin fixed and undergone chemical/heat dehydration and paraffin‐embedding. The staining intensity and percentage of positive cancer cells in all slides were scored according to the semi‐quantitative scoring system, which was independently performed by two experienced pathologists in the absence of clinical information. The score was calculated according to the staining intensity and the percentage of positive tumor cells. The scores were as follows: A, staining intensity: 0 (no staining), 1 (light staining), 2 (moderate staining), 3 (strong staining); B, the percentage of positive cells: 0 (< 5%), 1 (5%–25%), 2 (26%–50%), 3 (51%–75%), 4 (76%–100%). The total score of immunohistochemical staining = staining intensity × percentage of positive tumor cells. Our research has been approved by the Committee of the First Affiliated Hospital of Guangxi Medical University. All patients signed a written informed consent form for the use of their samples for research. The research method complies with the standards set by the ‘Declaration of Helsinki’.

### In‐house RNA‐seq

ESCC tissues and adjacent normal esophageal tissues from eight patients, six women, and two men, with ESCC who received treatment at the First Affiliated Hospital of Guangxi Medical University (Nanning, China) were collected, from April to August 2019. None of the patients had undergone preoperative chemotherapy or radiotherapy. The tissue samples were stored at −80 °C immediately after resection. Before the sequencing experiment, all the tissues were evaluated under the microscope in HE slices, and only the tissues with more than 80% ESCC tumor cells could be included. The specific methods and reagents for RNA‐seq have been described in detail by our team in previous articles [44]. In the current study, we obtained log 2 (x + 1) level 3 records of ESCC tissues and adjacent normal esophageal tissues from these eight patients with ESCC and obtained RNA‐seq data per million reads (TPM). Our research has been approved by the Committee of the First Affiliated Hospital of Guangxi Medical University. All patients signed a written informed consent form for the use of their samples for research. The research method complies with the standards set by the ‘Declaration of Helsinki’.

### Acquisition of external RNA‐seq and microarray data

The TCGA database is a large cohort platform that contains over 30 human tumors. The Genotype‐Tissue Expression database (GTEx) collects the RNA‐seq data of normal tissues. The external RNA‐seq data of esophageal cancer and control group were downloaded from the TCGA database and GTEx database. The data of ESCC were subsequently extracted separately for this study. A combination of the two databases not only yields the RNA‐seq data of ESCC and control group in the TCGA database, but also obtains the RNA‐seq data of normal esophageal tissue in the GTEx database, which expands the data information of RNA‐seq. In addition, we searched the GEO database and ArrayExpress database with the following keywords: “esophagus” or “esophageal” and “carcinoma” or “tumor” or “cancer” or “neoplas”* or “maliganan”*. The inclusion criteria were as follows: (a) the CDK1 expression profiles in ESCC and non‐ESCC controls tissues could be achieved, and (b) more than three tumors and controls were enrolled in the study.

The outline of this paper is depicted in Fig. S2.

### Integrated analysis and statistical methods

After log2(x + 1) conversion of the expression spectrum data by Excel 2016, spss 22.0 (Statistical Product and Service Solutions, IBM, Stanford, CA, USA) was used to calculate the number of samples (N), mean (M), standard deviation (SD) of ESCC and control group in each data set. Then, the difference in the expression level of CDK1 in ESCC and the control group was compared by Student's *t* test. The scatter plot / boxplot and receiver operating characteristic curve (ROC) were drawn using graphpad prism 8.0 (GraphPad Software, San Diego, CA, USA) to visualize the differences between the two groups. In addition, the standardized mean difference (SMD) forest plot was drawn using meta 4.15‐1 package of R3.6.3. SMD > 0, and *P* < 0.05 was considered as up expression; on the contrary, SMD < 0 and *P* < 0.05 was considered as down expression. To further evaluate the discriminating ability of CDK1 in ESCC and the control group, Metadisc 1.4 (Ramón y Cajal Hospital, Madrid, Spain) was used to draw the sROC curve and measure sensitivity, specificity, negative and positive likelihood ratio, and the diagnostic ODS ratio. Finally, the publication bias was detected using the funnel plot and the Egger’s and Begg’s tests. All statistical analyses were based on *P* < 0.05.

### Expression of CDK1 in cell lines

We searched the Cancer Cell Line Encyclopedia (CCLE) database for the keyword “*CDK1*,” and the mRNA expression profile data of ESCC cell lines were downloaded. Subsequently, the scatter plot of *CDK1* expression in different ESCC cell lines was constructed by Excel 2016.

### Differentially expressed RNA and CEGs associated CDK1

The annotation document of the corresponding platform was used to annotate the gene expression profile. If a gene corresponds to multiple probes, we took its average value as its gene expression value. The raw data were first standardized using the log2(x + 1) method and then normalized using the limma 3.42.2 package. Subsequently, the Limma 3.42.2 (Bioconductor, USA) package was used to process microarray data, and edger 3.28.1 (Bioconductor) package was used for RNA‐seq data. The screening criteria of differentially expressed gene was *P* < 0.05 and |logFC| > 1. The CEGs of CDK1 were screened according to the Pearson correlation coefficient using the R 3.6.3. When the correlation Pearson *R* > 0.5 and *P* < 0.05, it was considered as a co‐expressed gene. In addition, RobustRankAggreg (RRA) combined with artificial ranking was used to further screen the main CEGs associated CDK1. Then, VENNY2.1.0 (Spanish National Biotechnology Centre [CNB], Madrid, Spain) was used to intersect the CEGs and DEGs and draw a Venn diagram.

### Potential molecular mechanisms of CDK1 in ESCC

In order to investigate the molecular mechanism of CDK1 in ESCC, GO annotations and KEGG pathway enrichment of CDK1 CEGs and DEGs were analyzed using the clusterprofiler 3.14.3 package (Bioconductor). A PPI network was constructed using STRING website and cytoscape 3.7.1 (Institute for Systems Biology, Seattle, WA 98103, USA). Then, the MCODE algorithm was used to determine the hub genes in the neighborhood of protein dense connection.

### LncRNA‐miRNA‐CDK1 network construction

The miRNAs upstream of *CDK1* were predicted on the miRwalk 2.0, and these miRNAs were intersected with down‐regulated miRNAs in VENNY2.1.0 to obtain overlapping miRNAs of both. Then, the SMD of these overlapping miRNAs was calculated, and the low‐expression miRNAs with SMD < 0 and *P* < 0.05 were selected. The low‐expression miRNAs were input into the miRNet 2.0 database to predict the upstream lncRNAs. Similarly, the upstream lncRNAs were overlapped with the up‐regulated lncRNAs from the microarray and RNA‐seq data. Then, the SMD of these overlapping lncRNAs were calculated. Finally, lncRNAs with SMD > 0 and *P* < 0.05 were collected as highly expressed lncRNAs. The above miRNAs, lncRNAs, and CDK1 were input into the cytoscape 3.7.1 software to obtain the lncRNA‐miRNA‐*CDK1* ceRNA network.

## Conflict of interest

The authors declare no conflict of interest.

## Author contributions

HJZ conceived and designed the experiments, performed the experiments, analyzed the data, prepared figures and/or tables, authored or reviewed drafts of the paper, and approved the final draft. GC, SWC, and ZWF conceived and designed the experiments, performed the experiments, prepared figures and/or tables, authored or reviewed drafts of the paper, and approved the final draft. HFZ, ZBF, JXM, CBL, and JL conceived and designed the experiments, authored or reviewed drafts of the paper, and approved the final draft.

## Supporting information


**Fig S1.** The flow chart of this study. Note: DEGs, differentially expressed genes; CEGs, co‐expressed genes; GO, gene ontology; KEGG, Kyoto Encyclopedia of genes and genomes; PPI, protein interaction network; ceRNA, endogenous competitive RNA network.

**Fig S2.** Graphical representation of data collection process in this study. Note: GEO, Gene Expression Omnibus database; DEGs, differentially expressed genes; TCGA, The Cancer Genome Atlas Program; GTEx, The Genotype‐Tissue Expression.

**Table S1.** Relationship between CDK1 protein expression and clinicopathological factors in ESCC patients based on in‐house IHC.

**Table S2.** Relationship between CDK1 expression and clinicopathological parameters in ESCC based on external RNA‐seq data.

**Table S3.** The DEGs of the in‐house RNA‐seq.

**Appendix S1.** RScript for CEGs.

**Appendix S2.** RScript for DEG of microarray.

**Appendix S3.** RScript for DEG of RNA‐seq.

## Data Availability

The analyzed data sets generated during the present study are available from the corresponding author on reasonable request. The following information was supplied regarding data availability: The in‐house RNA‐seq data presented in the study are deposited in the NCBI Gene Expression Omnibus (GEO) (https://www.ncbi.nim.nih.gov/geo/) repository, accession number (GSE164158). The external RNA‐seq data can be obtained from The Cancer Genome Atlas (TCGA). Microarray data can be obtained from NCBI GEO: GSE45670, GSE77861, GSE26886, GSE69925, GSE100942, GSE33810, GSE17351, GSE20347, GSE38129, GSE29001, GSE33426, GSE23400, GSE70409, GSE45168, GSE77531, GSE71411, GSE111011, GSE53625, GSE66274, GSE75241, GSE32424.
