# Supplementary figures and images for: Overexpression of cyclin‐dependent kinase 1 in esophageal squamous cell carcinoma and its clinical significance
Source: FEBS Open Bio. 2021 Oct 19;11(11):3126–41. doi: 10.1002/2211-5463.13306 (PMC8564100; doi:10.1002/2211-5463.13306)

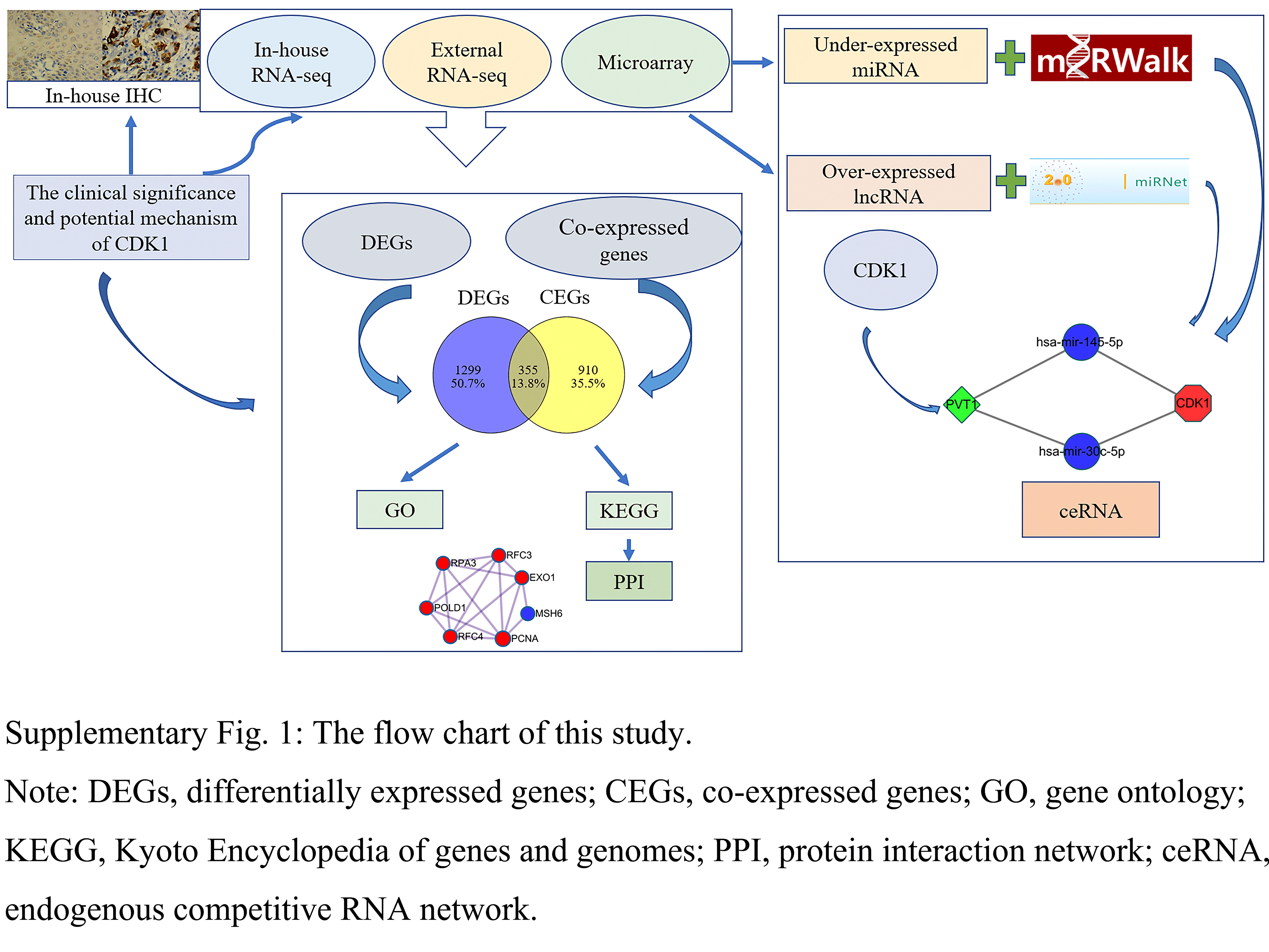

Supplement: Supplementary file 1 — Fig S1. The flow chart of this study. Note: DEGs, differentially expressed genes; CEGs, co‐expressed genes; GO, gene ontology; KEGG, Kyoto Encyclopedia of genes and genomes; PPI, protein interaction network; ceRNA, endogenous competitive RNA network. [file FEB4-11-3126-s005.tif]

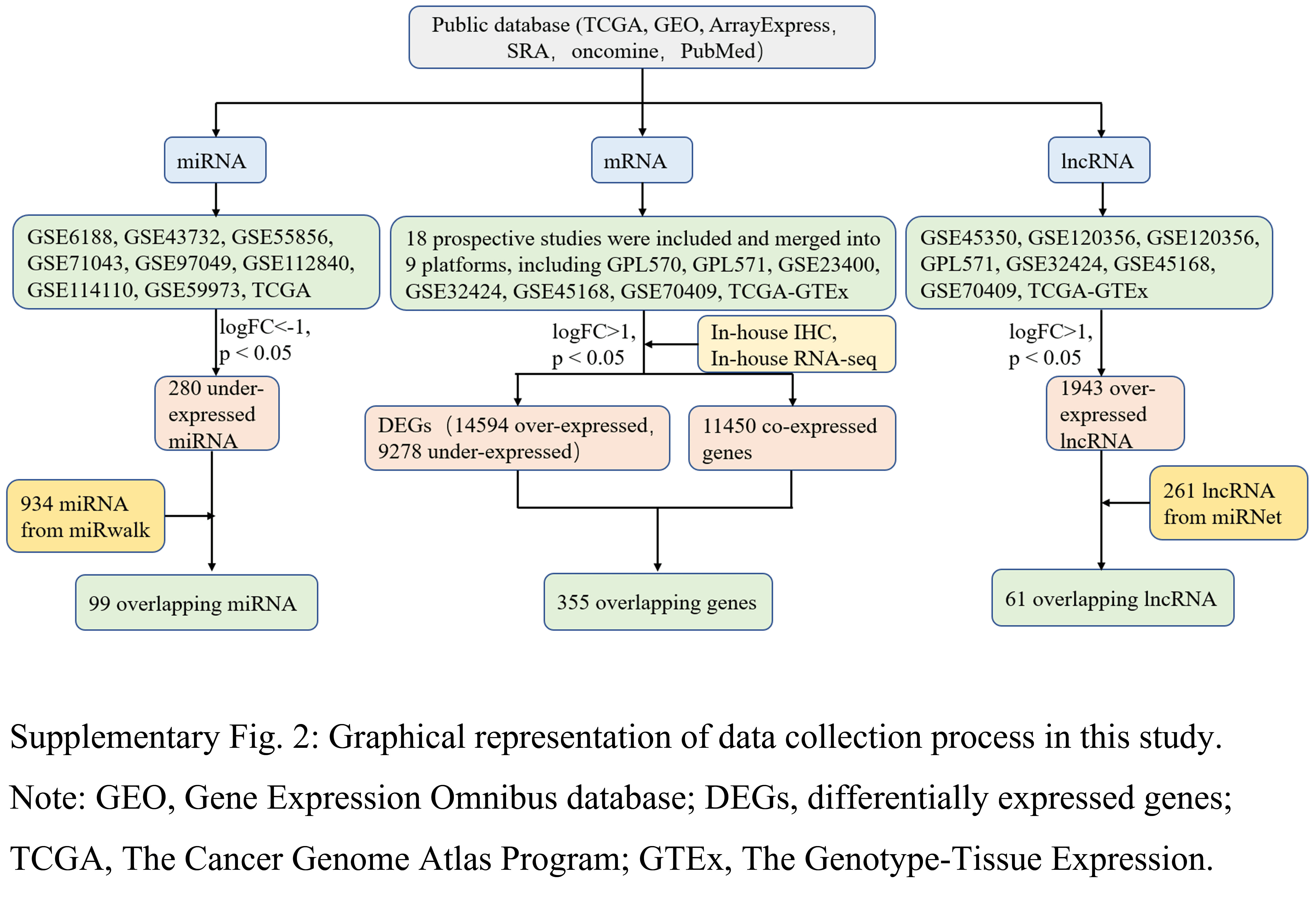

Supplement: Supplementary file 2 — Fig S2. Graphical representation of data collection process in this study. Note: GEO, Gene Expression Omnibus database; DEGs, differentially expressed genes; TCGA, The Cancer Genome Atlas Program; GTEx, The Genotype‐Tissue Expression. [file FEB4-11-3126-s004.tif]
