# Supplementary material for: Overexpression of cyclin‐dependent kinase 1 in esophageal squamous cell carcinoma and its clinical significance
Source: FEBS Open Bio. 2021 Oct 19;11(11):3126–41. doi: 10.1002/2211-5463.13306 (PMC8564100; doi:10.1002/2211-5463.13306)
Supplement: Supplementary file 3 — Table S1. Relationship between CDK1 protein expression and clinicopathological factors in ESCC patients based on in‐house IHC. [file FEB4-11-3126-s007.docx]

Supplementary Table 1:

Relationship between CDK1 protein expression and clinicopathological factors in ESCC patients.

| Parameters | N | Mean | SD | T | P-value |
| --- | --- | --- | --- | --- | --- |
| Tissue |  |  |  | -9.076 | <0.001 |
| Normal | 138 | 1.040 | 1.632 |  |  |
| Tumor | 151 | 3.540 | 2.923 |  |  |
| Gender |  |  |  | 0.102 | 0.919 |
| Female | 13 | 3.620 | 2.468 |  |  |
| Male | 138 | 3.530 | 2.970 |  |  |
| Age |  |  |  | 0.721 | 0.472 |
| ≤60 | 94 | 3.670 | 2.788 |  |  |
| >60 | 57 | 3.320 | 3.146 |  |  |
| Pathologic T stage |  |  |  | 0.994 | 0.322 |
| I-II | 49 | 3.880 | 2.870 |  |  |
| III-IV | 102 | 3.370 | 2.948 |  |  |
| Pathologic N stage |  |  |  | 1.010 | 0.314 |
| N0 | 71 | 3.690 | 3.254 |  |  |
| N1-N3 | 80 | 3.400 | 2.608 |  |  |
| Pathologic TNM stage |  |  |  | 1.349 | 0.179 |
| I-II | 85 | 3.810 | 3.145 |  |  |
| III-IV | 66 | 3.180 | 2.589 |  |  |
| Lymph node metastasis |  |  |  | -0.678 | 0.504 |
| NO | 17 | 0.350 | 0.493 |  |  |
| YES | 8 | 0.500 | 0.535 |  |  |
| Smoking |  |  |  | 0.979 | 0.338 |
| NO | 17 | 0.710 | 0.470 |  |  |
| YES | 8 | 0.500 | 0.535 |  |  |
| Alcohol |  |  |  | 1.316 | 0.201 |
| NO | 17 | 0.760 | 0.437 |  |  |
| YES | 8 | 0.500 | 0.535 |  |  |
