# Supplementary material for: Overexpression of cyclin‐dependent kinase 1 in esophageal squamous cell carcinoma and its clinical significance
Source: FEBS Open Bio. 2021 Oct 19;11(11):3126–41. doi: 10.1002/2211-5463.13306 (PMC8564100; doi:10.1002/2211-5463.13306)
Supplement: Supplementary file 4 — Table S2. Relationship between CDK1 expression and clinicopathological parameters in ESCC based on external RNA‐seq data. [file FEB4-11-3126-s003.docx]

Supplementary Table 2:

Relationship between CDK1 expression and clinicopathological parameters in ESCC based on external RNA-seq data.

| Parameters | N | Mean | SD | T | P-value |
| --- | --- | --- | --- | --- | --- |
| Tissue |  |  |  | -42.630 | <0.001 |
| Normal | 1456 | 7.306 | 2.484 |  |  |
| Tumor | 82 | 12.075 | 0.824 |  |  |
| Age |  |  |  | 0.459 | 0.001 |
| ≤60 | 52 | 12.303 | 0.748 |  |  |
| >60 | 29 | 11.681 | 0.826 |  |  |
| Gender |  |  |  | 1.099 | 0.275 |
| Female | 12 | 11.838 | 0.646 |  |  |
| Male | 69 | 12.122 | 0.852 |  |  |
| Pathologic T stage |  |  |  | -0.608 | 0.545 |
| I-II | 36 | 12.018 | 0.838 |  |  |
| III-IV | 45 | 12.130 | 0.825 |  |  |
| Pathologic N stage |  |  |  | 0.806 | 0.423 |
| N0 | 43 | 12.140 | 0.878 |  |  |
| N1-N3 | 30 | 11.975 | 0.844 |  |  |
| Pathologic M stage |  |  |  | -0.368 | 0.714 |
| M0 | 70 | 12.069 | 0.835 |  |  |
| M1 | 5 | 12.211 | 0.781 |  |  |
| Pathologic TNM stage |  |  |  | 0.260 | 0.796 |
| I-II | 54 | 12.097 | 0.816 |  |  |
| III-IV | 26 | 12.045 | 0.883 |  |  |
| Alcohol |  |  |  | -0.239 | 0.812 |
| NO | 19 | 12.030 | 0.743 |  |  |
| YES | 60 | 12.083 | 0.865 |  |  |
