# Supplementary material for: Overexpression of cyclin‐dependent kinase 1 in esophageal squamous cell carcinoma and its clinical significance
Source: FEBS Open Bio. 2021 Oct 19;11(11):3126–41. doi: 10.1002/2211-5463.13306 (PMC8564100; doi:10.1002/2211-5463.13306)
Supplement: Supplementary file 5 — Table S3. The DEGs of the in‐house RNA‐seq. [file FEB4-11-3126-s008.docx]

Supplementary Table 3:

The DEGs of the in-house RNA-seq.

| Gene | Log 2 | P-value | FDR |
| --- | --- | --- | --- |
| MMP1 | 3.058 | 4.97E-12 | 9.45E-08 |
| MMP3 | 3.920 | 2.84E-09 | 1.92E-05 |
| CST1 | 3.310 | 3.02E-09 | 1.92E-05 |
| MMP10 | 2.843 | 1.37E-08 | 5.67E-05 |
| AC084879 | -4.797 | 1.49E-08 | 5.67E-05 |
| COL11A1 | 2.764 | 4.41E-08 | 0.00014 |
| HOXD10 | 2.899 | 5.65E-08 | 0.000154 |
| OR13H1 | 4.281 | 1.19E-07 | 0.000247 |
| IGLL4P | 2.957 | 1.23E-07 | 0.000247 |
| CRISP3 | -2.610 | 1.30E-07 | 0.000247 |
| HOXD11 | 3.510 | 4.17E-07 | 0.000698 |
| CSAG3 | 4.004 | 4.70E-07 | 0.000698 |
| AC024581 | 4.497 | 4.77E-07 | 0.000698 |
| BPIFB2 | -2.561 | 5.40E-07 | 0.000734 |
| MMP12 | 2.408 | 9.43E-07 | 0.001196 |
| MAGEA4 | 3.948 | 1.04E-06 | 0.001233 |
| RHOT1P2 | -2.983 | 1.61E-06 | 0.001638 |
| AL356867 | -2.860 | 1.61E-06 | 0.001638 |
| RHOT1P1 | -2.369 | 1.64E-06 | 0.001638 |
| AC002075 | 2.349 | 1.83E-06 | 0.001742 |
| MMP13 | 2.707 | 1.94E-06 | 0.001759 |
| HOXC10 | 2.932 | 2.56E-06 | 0.002161 |
| MUC5B | -1.930 | 2.61E-06 | 0.002161 |
| CYCSP6 | 3.934 | 3.27E-06 | 0.002463 |
| MAGEA2B | 3.274 | 3.27E-06 | 0.002463 |
| SNX18P13 | -2.391 | 3.37E-06 | 0.002463 |
| MYOC | -2.209 | 5.30E-06 | 0.003734 |
| KRT75 | 3.281 | 5.65E-06 | 0.003837 |
| OR13K1P | 3.854 | 7.63E-06 | 0.004861 |
| ADH1B | -1.830 | 7.67E-06 | 0.004861 |
| POTEE | 3.684 | 8.11E-06 | 0.004977 |
| CARSP2 | 2.816 | 8.43E-06 | 0.005012 |
| AL805909 | -2.727 | 9.42E-06 | 0.005429 |
| BMS1P13 | -3.698 | 1.10E-05 | 0.00549 |
| AL022100 | 3.431 | 1.10E-05 | 0.00549 |
| AP005212 | -3.212 | 1.10E-05 | 0.00549 |
| AC008885 | 3.208 | 1.10E-05 | 0.00549 |
| AL359711 | 3.152 | 1.10E-05 | 0.00549 |
| AC010677 | 3.388 | 1.18E-05 | 0.005772 |
| MMP9 | 1.888 | 1.40E-05 | 0.006502 |
| ATP1A2 | -1.810 | 1.40E-05 | 0.006502 |
| AL080285 | 4.167 | 1.53E-05 | 0.006751 |
| AC079448 | -4.136 | 1.53E-05 | 0.006751 |
| CYP4F29P | -2.014 | 1.81E-05 | 0.007825 |
| SFTA2 | -3.090 | 2.11E-05 | 0.008937 |
| MMP11 | 1.567 | 2.17E-05 | 0.00897 |
| IL36A | -1.641 | 3.05E-05 | 0.011848 |
| POTEF | 3.894 | 3.05E-05 | 0.011848 |
| DSPP | 3.566 | 3.05E-05 | 0.011848 |
| MAL | -1.344 | 3.11E-05 | 0.011849 |
| IGHJ4 | -4.105 | 3.20E-05 | 0.011942 |
| MYZAP | -1.806 | 3.31E-05 | 0.012093 |
| AC026700 | 2.795 | 3.59E-05 | 0.012879 |
| AC090897 | -2.766 | 3.76E-05 | 0.013243 |
| ASB5 | -1.844 | 4.09E-05 | 0.014158 |
| MAGEA3 | 3.536 | 4.26E-05 | 0.014468 |
| SPINK7 | -1.602 | 4.65E-05 | 0.01545 |
| C2orf40 | -1.637 | 4.71E-05 | 0.01545 |
| LINC00328-2P | -2.143 | 4.83E-05 | 0.015576 |
| MUC21 | -1.555 | 5.58E-05 | 0.017691 |
| PCP4 | -1.562 | 5.78E-05 | 0.018021 |
| LRRC15 | 2.831 | 6.60E-05 | 0.019986 |
| KREMEN2 | 2.217 | 6.62E-05 | 0.019986 |
| AC018865 | 3.056 | 7.07E-05 | 0.021028 |
| SCGB3A1 | -2.119 | 7.78E-05 | 0.022771 |
| CSAG1 | 3.460 | 8.06E-05 | 0.023224 |
| SPP1 | 1.310 | 8.72E-05 | 0.024724 |
| PPP1R3C | -1.545 | 8.84E-05 | 0.024724 |
| KRT17 | 1.009 | 9.21E-05 | 0.025385 |
| PI16 | -1.746 | 9.85E-05 | 0.026766 |
| ESM1 | 2.424 | 0.000104 | 0.02774 |
| CRNN | -1.209 | 0.000105 | 0.02774 |
| BMS1P17 | 1.920 | 0.000112 | 0.029068 |
| HOXC9 | 2.159 | 0.000113 | 0.029068 |
| DPCR1 | -3.079 | 0.000121 | 0.030316 |
| CXCL11 | 2.790 | 0.000121 | 0.030316 |
| AMTN | 2.182 | 0.000124 | 0.03067 |
| CYP4A27P | -3.244 | 0.000145 | 0.034906 |
| LGR5 | 3.147 | 0.000145 | 0.034906 |
| SCGB2A2 | -3.340 | 0.000152 | 0.035687 |
| FLG | -1.763 | 0.000156 | 0.035687 |
| CXCL10 | 1.741 | 0.000156 | 0.035687 |
| ZNF536 | -2.435 | 0.000157 | 0.035687 |
| C7 | -1.173 | 0.000158 | 0.035687 |
| BPIFB1 | -1.649 | 0.000164 | 0.036626 |
| KRT24 | -2.756 | 0.000168 | 0.036672 |
| CYP4F34P | -2.756 | 0.000168 | 0.036672 |
| AC012501 | 2.414 | 0.00018 | 0.03891 |
| SALL4 | 2.178 | 0.000192 | 0.040625 |
| AL355796 | 2.162 | 0.000192 | 0.040625 |
| SCARA5 | -1.648 | 0.000198 | 0.0414 |
| TFF3 | -1.814 | 0.000204 | 0.042127 |
| CTHRC1 | 1.315 | 0.000217 | 0.044305 |
| CFL1P7 | -2.713 | 0.000221 | 0.044305 |
| AL031736 | 2.626 | 0.000221 | 0.044305 |
